# Women’s Health Study (WENDY)—a protocol of a population-based study assessing gynecological and metabolic health in women in their mid-30s

**DOI:** 10.1093/aje/kwae230

**Published:** 2024-08-03

**Authors:** Terhi T Piltonen, Maria Ohtamaa, Riikka K Arffman, Lotta Vuokila, Elisa Hurskainen, Minna Männikkö, Laura Huilaja, Suvi-Päivikki Sinikumpu, Tero Rautio, Katariina Kilpivaara, Jari Jokelainen, Eetu Kiviniemi, Pekka Pinola, Minna Törnävä, Elina Komsi, Marika H Kangasniemi, Maria Rajecki, Kaisu Luiro, Jenni Kinnunen, Susanna M Savukoski

**Affiliations:** Department of Obstetrics and Gynecology, Research Unit of Clinical Medicine, Medical Research Centre, University of Oulu, Oulu University Hospital, Oulu, Finland; Department of Obstetrics and Gynecology, Research Unit of Clinical Medicine, Medical Research Centre, University of Oulu, Oulu University Hospital, Oulu, Finland; Department of Obstetrics and Gynecology, Research Unit of Clinical Medicine, Medical Research Centre, University of Oulu, Oulu University Hospital, Oulu, Finland; Department of Obstetrics and Gynecology, Research Unit of Clinical Medicine, Medical Research Centre, University of Oulu, Oulu University Hospital, Oulu, Finland; Department of Obstetrics and Gynecology, Research Unit of Clinical Medicine, Medical Research Centre, University of Oulu, Oulu University Hospital, Oulu, Finland; Northern Finland Birth Cohorts, Arctic Biobank, Infrastructure for Population Studies, Faculty of Medicine, University of Oulu, Oulu, Finland; Department of Dermatology, Research Unit of Clinical Medicine, Medical Research Centre, University of Oulu, Oulu University Hospital, Oulu, Finland; Department of Dermatology, Research Unit of Clinical Medicine, Medical Research Centre, University of Oulu, Oulu University Hospital, Oulu, Finland; Department of Surgery, Research Unit of Translational Medicine, Medical Research Centre, University of Oulu, Oulu University Hospital, Oulu, Finland; Department of Surgery, Research Unit of Translational Medicine, Medical Research Centre, University of Oulu, Oulu University Hospital, Oulu, Finland; Northern Finland Birth Cohorts, Arctic Biobank, Infrastructure for Population Studies, Faculty of Medicine, University of Oulu, Oulu, Finland; Northern Finland Birth Cohorts, Arctic Biobank, Infrastructure for Population Studies, Faculty of Medicine, University of Oulu, Oulu, Finland; Department of Obstetrics and Gynecology, Research Unit of Clinical Medicine, Medical Research Centre, University of Oulu, Oulu University Hospital, Oulu, Finland; Social Services and Health Care; Tampere University of Applied Sciences, Kuntokatu, Tampere, Finland; Department of Obstetrics and Gynecology, Research Unit of Clinical Medicine, Medical Research Centre, University of Oulu, Oulu University Hospital, Oulu, Finland; Department of Obstetrics and Gynecology, Research Unit of Clinical Medicine, Medical Research Centre, University of Oulu, Oulu University Hospital, Oulu, Finland; Department of Obstetrics and Gynaecology, Reproductive Medicine Unit, Helsinki University Hospital, University of Helsinki, Helsinki, Finland; Department of Obstetrics and Gynaecology, Reproductive Medicine Unit, Helsinki University Hospital, University of Helsinki, Helsinki, Finland; Department of Obstetrics and Gynecology, Research Unit of Clinical Medicine, Medical Research Centre, University of Oulu, Oulu University Hospital, Oulu, Finland; Department of Obstetrics and Gynecology, Research Unit of Clinical Medicine, Medical Research Centre, University of Oulu, Oulu University Hospital, Oulu, Finland

**Keywords:** birth cohort, women’s health, population-based study, reproductive health, metabolic health

## Abstract

The Women’s Health Study (WENDY) was conducted to improve insights into women’s health and health burden. It provides a unique, comprehensive data source that can be broadly utilized to understand gynecological symptoms, diseases, and their relation to metabolic and overall health more deeply in a population-based setting. The study was conducted in Finland from May 2020 to October 2022. It included 1918 women (33-37 years old) who were born in northern Finland between July 1985 and December 1987. Data collection comprised one 3- to 4-hour study visit that included clinical measurements, biological samples, ultrasound examinations and an extensive questionnaire on gynecological and reproductive history, physical and mental health, quality of life, lifestyles, current life situations, health awareness, and opinions. The study also included a menstrual cycle follow-up and cognitive testing up to 3 months via a mobile application. Given that all participants’ data can be linked to all Finnish national registers, and the Northern Finland Birth Cohort participants’ data can be linked to the birth cohort data set collected from gestational week 24 onward, WENDY study forms one of the largest data sets worldwide to investigate gynecological and metabolic health burden in women.

## Introduction

Several common gynecological conditions are poorly recognized in the health care system due to a lack of awareness, knowledge, and resources. Moreover, some women may be reluctant to report their symptoms due to social or cultural factors. Although many gynecological health impairments or diseases—such as heavy menstrual bleeding, polycystic ovary syndrome (PCOS), endometriosis, and vulvodynia—greatly affect quality of life (QoL),^1-3^ the delay between symptoms’ appearance and diagnosis or treatment is typically long.^4-6^ Moreover, metabolic derangements have become more prevalent in women, also affecting gynecological health and QoL.^2^^,7-9^ Fortunately, in recent years, public knowledge and the pressure to focus on female health issues have increased alongside health equity in many countries worldwide.^10-14^ Moreover, the pharmaceutical industry has become more aware of women’s gynecological and reproductive health issues, as well as such conditions’ financial implications, enabling the expansion and targeting of resources for this population.^15^

The Women’s Health Study (WENDY) was intended to comprehensively investigate women’s overall health while focusing on gynecological and metabolic health during fertile ages. Previously, several cohort studies have involved female participants, such as the Nurses’ Health Study (United States),^16^ the Australian Longitudinal Study on Women’s Health,^17^ the National Longitudinal Survey of Mature and Young Women (United States),^18^ and the Study of Women’s Health across the Nation (United States).^19^ However, to the best of our knowledge, no previous large studies based on female populations have focused specifically on gynecological issues, including the broad evaluation of metabolic factors, or allowed for register-data linkages on a great scale, from birth to the present.

WENDY data constitute the world’s largest data set targeting women’s reproductive and metabolic health. The data are available from the fetal stage onward and includes clinical measures, biological samples, and many questions and validated questionnaires evaluating physical and mental well-being, lifestyle, and opinions. In addition, the data may be linked to high-quality national Finnish registers.^20^ The WENDY data will be used comprehensively to investigate the prevalence of gynecological diseases and symptoms, as well as their association with overall health and well-being. The study was aimed to improve diagnostics and health awareness and aid in novel tool development.

## Methods

### Study population

The study focused on women in their mid-30s. WENDY’s main study population (population 1) was the participants in the prospective, population-based Northern Finland Birth Cohort 1986 (NFBC1986),^21^ which originally included 4567 women. The recruitment of NFBC1986 participants began in 1984, when all pregnant mothers living in Oulu and Lapland who were expected to deliver between July 1, 1985, and June 30, 1986, were invited to participate. This prospective birth cohort’s data have been comprehensively collected since their antenatal period at 24 gestational weeks, including at delivery, from 1 month to 6 years​ of age via child welfare clinics, and at the ages of 7 to 8, 15 to 16, and 33 to 35 years via NFBC1986 follow-up studies.^21^^,^^22^ The collected data include clinical examinations, biological samples, imaging studies, and questionnaires. Biological samples are available for patients’ 15- to 16-year-old and 33- to 35-year-old data. The gynecological health of the participating young women and their mothers was studied when the participants were 26 years old using a questionnaire. All living female participants of NFBC1986 with known addresses in Finland on January 1, 2020 (*n* = 4292) were invited to participate in WENDY at Oulu or Helsinki sites between 2020 and 2022. During WENDY’s trial, these women were 33 to 37 years old. Of the invited women, 1544 (36%) participated in the WENDY trial.

Additionally, 1112 randomly selected women who had been born in the same area up to 1.5 years after the last birth in the NFBC1986 cohort (August 1, 1986, to December 31, 1987) and who lived within 100 km of Oulu were also invited to participate in WENDY. Of these 1112 women, 374 (34%) participated (population 2). During the WENDY trial, these women were 34 to 36 years old.

**Figure 1:**
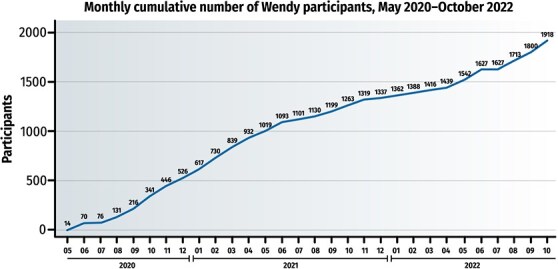
Monthly cumulative number of the WENDY participants.

**Figure 2:**
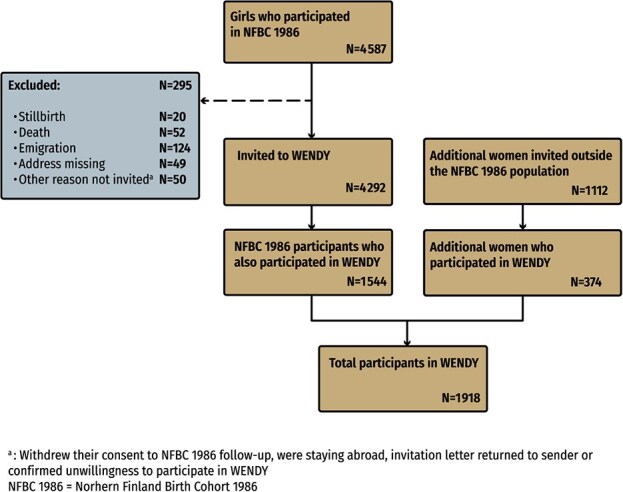
Flowchart of the study population in WENDY.

*Total population*. In total, 1918 women aged 33 to 37 years participated in WENDY. Of these women, 1879 (98%) permitted their data to be linked to the national registers.^6^ Moreover, 1544 (81%) of WENDY’s participants were participants of NFBC1986, which also enabled the linkage of the WENDY and NFBC1986 data sets. The WENDY participants’ basal demographics are presented in Table 1. The cumulative number of women who participated in the WENDY study by month is shown in Figure 1. Figure 2 presents a flowchart depicting the study’s population.

**Table 1:** Basal demographics of the WENDY study participants.

	**Population 1**	**Population 2**	**Total population**
	***n* = 1544**	***n* = 374**	***n* = 1918**
Age, mean (SD), y^a^	35.3 (0.6)	35.3 (0.4)	35.3 (0.6)
Body mass index, mean (SD)	25.9 (5.5)	26.5 (5.6)	26.0 (5.5)
Education, *n* (%)			
Basic or less	9 (0.6)	6 (1.6)	15 (0.8)
Secondary	455 (29.5)	122 (32.6)	577 (30.1)
Tertiary	1079 (69.9)	246 (65.8)	1325 (69.1)
Ethnicity, *n* (%)			
Caucasian	1503 (97.3)	369 (98.7)	1872 (97.6)
Sami	40 (2.6)	3 (0.8)	43 (2.2)
Other	1 (0.1)	2 (0.5)	3 (0.2)
Smoking, *n* (%)			
Never	763 (49.4)	176 (47.1)	939 (49.0)
No, previously yes	546 (35.4)	133 (35.6)	679 (35.4)
Yes, less than daily	110 (7.1)	27 (7.2)	137 (7.1)
Yes, daily	125 (8.1)	38 (10.2)	163 (8.5)
Current contraceptive method, *n* (%)			
Hormonal	546 (35.4)	134 (35.8)	680 (35.5)
Nonhormonal	592 (38.3)	157 (42.2)	749 (39.1)
Other	220 (14.2)	41 (11.0)	261 (13.6)
No contraception	341 (22.1)	70 (18.7)	411 (21.4)
Parity, *n* (%)			
Nulliparous women	463 (30.0)	76 (20.3)	539 (28.1)
Parous women	1081 (70.0)	298 (79.7)	1379 (71.9)

^a^At the data collection in 2020-2022.

### Data storage

During data collection, all data were entered into the secure REDCap (Research Electronic Data Capture) web platform, except the results of the laboratory measurements, photographs, and ultrasound images, which were stored on the University of Oulu’s secure servers. All other data were stored in the Oulu University Hospital’s secure data storage. Data collection was fully electronic except for participants’ consent forms, which were stored in the University of Oulu’s file storage. After the study’s completion, all data were transferred to and stored in the University of Oulu’s secure data storage and merged with the core NFBC data set. All biological samples were stored at −80°C in the NFBC’s core sample storage at the University of Oulu. Each participant was assigned a unique code, which was used in the study’s data and sample storage, and the data forms did not include any personal identifiers.

### Data collected

#### Invitations

All living NFBC1986 participants who were assigned female at birth with known addresses in Finland (*n* = 4292) were invited to the clinical study visit at either an Oulu or a Helsinki site, depending on their residence on January 1, 2020. These appointments were arranged in Oulu for May 2020 to October 2022 and in Helsinki for September 2020 to June 2021. The first study invitations were sent via mail to addresses obtained from the Digital and Population Data Services Agency. Additionally, 1 to 2 reminder letters were sent if no response to previous invitations was obtained. One reminder letter was sent to 3001 (70%) prospective participants, and 2 reminder letters were sent to 2449 (57%). When WENDY began, a 33- to 35-year follow-up study was ongoing of all NFBC1986 participants’ overall health and working ability, and WENDY advertisements were distributed to all participating women. The study was also advertised through social media, national newspapers, and advertisements around the Oulu and Helsinki areas. Occasional invitations were sent by telephone—for example, when a participant canceled her appointment without making a new reservation. Women in population 2 (*n* = 1112) were invited to the Oulu study site via 1 posted letter each.

The study’s invitation letters included information on WENDY’s purpose and study visits’ practicalities. For pregnant women, it was stated that the study visits should occur no earlier than 6 months after delivery. Invitees also received information about the examinations that would be performed during the study visits, data collection, and data storage. They were also asked to consent to an exchange of data and biological samples between Finland and other countries for scientific research purposes. Separate consent was also solicited to provide genetic data and possibly participate in daily cognitive testing (by Cambridge Cognition Ltd) or menstrual cycle tracking for the next 3 months.

The participants were practically instructed to fast for 12 hours before their study visits since some blood parameters, body composition measurements (Inbody), and liver transient elastography (FibroScan) required fasting. Participants were also informed not to wear any makeup or jewelry to allow for body composition measurements and skin evaluations. The letters listed which data participants would be provided at or after their study visits (Table 2). A questionnaire regarding general health, medical history (especially gynecological and obstetric history and symptoms, as well as the dates of 3 last menstruations), and lifestyles was attached for participants to fill out before their study visits to facilitate their interviews with the study’s nurses.

**Table 2:** Data provided to each WENDY study participant during and after their study visit.

Gynecological ultrasonography findings
Abdominal ultrasonography: results of interrectus distance measurements
Results of transient elastography of the liver
Diastolic and systolic blood pressure
Summary of the body composition measurements
Weight, height, and body mass index
Waist and hip circumferences
Results of skin sebum measurement
Moona^a^ application: menstrual cycle and related symptoms (access to self-reported data)
Blood sampling results^b^
Results of finger-test assessment of pelvic floor muscle strength

^a^ https://www.moona.info/en

^b^Complete blood count, ferritin, lipid profile, parameters evaluating liver and kidney function, and parameters evaluating glucose metabolism and high-sensitivity C-reactive protein.

The participants were instructed to book their study visits through the NFBC1986 Women’s Health Study webpage electronic calendar using a unique code attached to each participant’s invitation letter or by email or telephone. The study visits and parking (at Oulu) were free of charge. The population 1 participants received travel compensation of 50 euros for their study visits from October 2021 onward, which is in line with the Finnish legislation.

#### Study visits’ process

The study visits lasted, on average, 3 hours. They comprised clinical examinations, interviews, biological sampling, and a self-reported questionnaire. In Oulu, the onsite personnel comprised a doctor and 3 nurses or midwives vs 1 doctor and 2 nurses or midwives in Helsinki. The doctors were specialists or experienced trainees in obstetrics and gynecology. Throughout the study, 6 nurses or midwives and 5 doctors took part in data collection. All staff members obtained Good Clinical Practice certification^23^ before WENDY.

All women who attended the study visits signed an informed consent form. The study visits started with anthropometric and blood pressure measurements and blood sampling. Gynecological histories were recorded, and the gynecologist performed clinical examinations. Next, the participants received breakfast and completed an electronic questionnaire. At the end of the visits, the nurses or midwives performed interviews and short clinical examinations. Additionally, the participants completed a short feedback questionnaire before leaving.

#### Interviews

During the visits, the gynecologist’s interview discussed the participants’ gynecological histories. The nurses and midwives interviewed the participants using the questionnaires the participants had already completed with their invitation letters. The questionnaire’s topics are presented in Table 3.

**Table 3:** Questions and topics in the interview and self-reported questionnaires.

Highest level of education
Lifestyle (tobacco, snuff, alcohol use)
Handedness
Current chronic diseases
History of cancer and cancer treatments
Age at menarche
Natural menstrual cycle history (beginning dates of the 3 last menstrual cycles)
Use of different contraceptive methods (current and past)
Pregnancies (deliveries, stillbirths, miscarriages, pregnancy terminations, extrauterine pregnancies)
Current status of breastfeeding
Infertility evaluations and possible treatments
Gynecological diseases^a^
Gynecological and abdominal operations
Familiarity with different women’s health issues or conditions^b^
Symptoms that might correlate with rectus diastasis
Bothersome hair growth (hirsutism) and hair loss; skin hair removal habits
36-Item Short Form Survey (SF-36)^32^^,^^33^
Experiences with and opinions about the COVID-19 pandemic
Stunkard’s Figure Rating Scale (FRS; self-assessment and ideal body image)^34^^,^^35^
Body Image Concern Inventory (BICI)^36^
Female Sexual Function Index (FSFI; with questions from 6- and 9-item versions of the FSFI questionnaires)^37-39^
Women’s Health Questionnaire (WHQ)^40^
Evaluation of vulvar pain–related symptoms
Desire for pregnancy
Evaluation of factors affecting the desire for pregnancy
History and experiences with breastfeeding
Experiences and opinions concerning menstruation
Evaluation of sleeping disorders
Evaluation of heavy menstrual bleeding
Bleeding assessment tool^41^
Evaluation of endometriosis-related symptoms
Awareness of several important facts related to female reproductive health
Evaluation of sex life–related issues and factors that affect sexual well-being
The self-rated control of pelvic floor muscles and their training habits

^a^Polycystic ovarian syndrome or polycystic ovaries seen with ultrasound, endometriosis, vulvodynia, stress or urge incontinence, recurrent vaginal candidiasis or bacterial infections, genital herpes, condyloma, and recurrent urinary tract infections.

^b^Polycystic ovarian syndrome, endometriosis, vulvodynia, stress or urge incontinence, human papillomavirus, condyloma, abdominal rectus diastasis, and early menopause.

#### Clinical examinations

**Figure 3:**
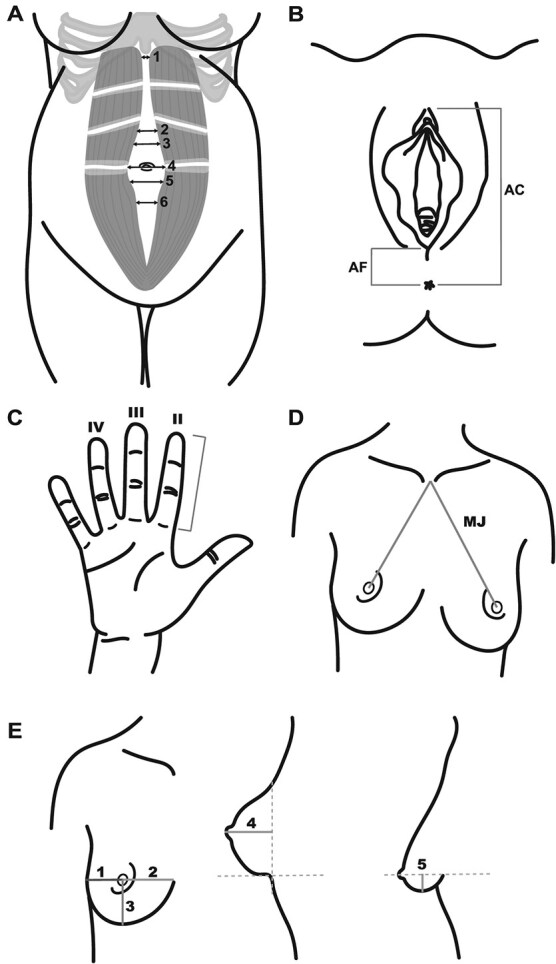
Diastasis recti, anogenital distance, and finger and breast measurements in WENDY. The diastasis measurements were obtained from 6 different anatomic sites (A). Table 4 presents a more detailed description of the measurements. The anogenital distance was measured from 2 different sites (B). The lengths of 3 different fingers were measured (C). Breast measurements are shown in D and E. AC, anus to clitoris; AF, anus to posterior fourchette; MJ, mamille-jugulum distance.

The measurements obtained by the gynecologist, nurses, and midwives are presented in Table 4. Interrectus distance, anogenital distance, finger length, and breast measurements are visualized in Figure 3.

**Table 4:** Measurements and other clinical examinations at the study visit.

**Measurement**	**No. of repeated measurements**	**Unit**	**Additional information**	**Measurement instrument**	**No. (%) of study participants measured**
Weight	1	kg		Bioelectrical impedance analyzer	1915 (99.8)
Height	2	cm		Measuring tape	1917 (99.9)
Blood pressure (systolic and diastolic)	3	mm Hg	Omron 10 blood pressure monitor	Automated oscillometric blood pressure device	1915 (99.8)
Neck circumference	2	cm		Measuring tape	1657 (86.4)
Hip circumference	1	cm		Measuring tape	1912 (99.7)
Waist circumference	1	cm		Measuring tape	1915 (99.8)
Body composition	1	Various units	InBody 720	Bioelectrical impedance analyzer	1913 (99.7)
Interrectus distance measurements^42^^,^^43^		cm	Samsung HS60	Abdominal ultrasound, linear transducer	
Xiphoideum	1				1914 (99.8)
4.5 cm above the umbilicus	1	cm			1387 (72.3)
3 cm above the umbilicus	1	cm			1912 (99.7)
At the umbilicus level	1	cm			1380 (28.1)
2 cm below the umbilicus	1	cm			1913 (99.7)
4.5 cm below the umbilicus	1	cm			1388 (72.4)
The widest interrectus distance, if not in the previous areas	1	cm			852 (44.4)
Evaluation of liver steatosis and fibrosis	10	kPa/dB/m	Probes M and XL in use	Transient elastography (FibroScan 430 mini+	1903 (99.2)
Intercostal ultrasound clip of the liver and right kidney	1			Abdominal ultrasound (curvilinear transducer)	1913 (99.7)
Anogenital distances	2	mm	Two different measurements: anus to clitoris, anus to posterior fourchette	Digital vernier caliper	1910 (99.6)
The strength of the pelvic floor muscles^24^^,^^25^	3	24,25	Best of the 3 contractions	Vaginal palpation	1043 (54.4)
Gynecological ultrasonography			Samsung HS60		
Uterus					
Corpus	1	cm		Vaginal ultrasound	1906 (99.4)
Anterior wall thickness	1	cm		Vaginal ultrasound	1906 (99.4)
Posterior wall thickness	1	cm		Vaginal ultrasound	1906 (99.4)
Endometrium	3	cm		Vaginal ultrasound	1906 (99.4)
3D clip	1			Vaginal ultrasound	1906 (99.4)
Ovaries			Both ovaries measured	Vaginal ultrasound	1866 (97.3)
Length, width, height	1	cm		Vaginal ultrasound	1866 (97.3)
Volume	1	cm^3^		Vaginal ultrasound	1866 (97.3)
Antral follicle count	1		Counted by the gynecologist	Vaginal ultrasound	1874 (97.7)
3D clips and assessment of dominant follicle, corpus luteum, and other ovarian findings	1		Ovarian cyst (>25 mm), endometrioma, dermoid, hemorrhagic cyst, paraovarian cyst, myoma, adenomyosis, endometrial polyp, sacto, fluid in fossa Douglas, other specified or specified finding	Vaginal ultrasound	1899 (99.0)
Breast measurements	1	cm	The right breast was measured; Figure 3	Measuring tape	1583 (82.5)
Length of the II, III, IV fingers	2	cm	The right-hand fingers were measured; Figure 3	Digital vernier caliper	1913 (99.7)
Skin sebum measurement	2	Sebumeter value	Sebumeter SM 815 display device (MDD4) (Enviroderm Services) Measurements from both sides of the forehead	Sebum-sensitive film	1543 (80.4)
Grip strength	3		Both hands	Saehan (MVS in Motion) hydraulic hand dynamometer	1905 (99.3)
Skin photographs (face)	1			Digital camera	1867 (97.3)
Skin photographs (back)	1			Digital camera	1880 (98.0)
Breast photographs	1			Digital camera	1376 (71.7)
Ferriman-Gallway rating	1		Clinical estimation by the gynecologist		1917 (99.9)
Vaginal swab	1		Vaginal microbiota evaluation		1917 (99.9)
Blood samples	1		Table 5		1764 (92.1)

The nurses and midwives measured weight, height, body composition, and finger length and photographed participants’ faces, backs, and breasts for later evaluation by an experienced dermatologist and plastic surgery team. The breast measurements were obtained primarily from the right side. In cases of significantly different sizes between breasts, the left breast was also measured. If a participant was currently breastfeeding, breast photographs and measurements were not taken.

The gynecologist’s clinical examinations obtained measurements of the interrectus distance from the rectus abdominis via transabdominal ultrasonography, intercostal ultrasound clips of the liver and kidney to evaluate liver fat content, and transient elastography (FibroScan) to evaluate liver steatosis and fibrosis. Transvaginal gynecological ultrasounds obtained uterine and ovarian assessment and measurements and examined the pelvis overall. Two vaginal swabs were taken. The strength of the pelvic floor muscles was assessed via digital palpation using the Oxford grading scale,^24^^,^^25^ and the anogenital distance and perineal height were measured via a digital vernier caliper (Figure 3). Clinical assessments of hirsutism were conducted using the Ferriman-Gallwey score.^26^^,^^27^ The gynecologist photographed participants’ thigh and groin areas in cases of hidradenitis.

#### Questionnaires

The participants answered a questionnaire, which included several validated questionnaires and additional questions, which are presented in Table 3, using a tablet. Their answers were recorded directly to REDCap.

#### Biological samples

The nurses and midwives took fasting blood samples, which were analyzed for the hormonal and metabolic parameters listed in Table 5. Participants’ serum and plasma samples and 2 vaginal swab samples were stored at −80°C.

**Table 5:** Conducted biological sample analyses and stored biological samples of the WENDY study. All samples were taken after participants had fasted for at least 10 hours.

**Sample name**	**Abbreviation**	**Sample type**	**Measurement**
Alanine aminotransferase	ALAT	Plasma	Photometric, as recommended by IFCC
Albumin	ALB	Plasma	Photometric, bromocresol purple
Alkaline phosphatase	AFOS	Plasma	Photometric, as recommended by IFCC
Anti-Müllerian hormone	AMH	Serum	Electrochemiluminescence immunoassay
Amylase	AMY	Plasma	Photometric, as recommended by IFCC
Androstenedione	A4	Serum	Electrochemiluminescence immunoassay
Aspartate aminotransferase	ASAT	Plasma	Photometric, as recommended by IFCC
Blood glycosylated hemoglobin	HbA1c	Whole blood	Photometric, enzymatic
Cholesterol (HDL)	HDL-C	Plasma	Photometric, direct enzymatic
Cholesterol (LDL)	LDL-C	Plasma	Photometric, direct enzymatic
Cholesterol (total)	TC	Plasma	Photometric, enzymatic
Complete blood count	CBC	Whole blood	Automatic cell counting
C-peptide	C-PEP	Serum	Immunochemiluminometric
Creatinine	CREA	Plasma	Photometric, enzymatic
Ferritin	FER	Serum	Immunochemiluminometric
Follicle-stimulating hormone	FSH	Serum	Immunochemiluminometric
γ-Glutamyl transferase	GT	Plasma	Photometric, as recommended by IFCC
Glucose	GLU	Plasma	Photometric, enzymatic
High-sensitivity C-reactive protein	hs-CRP	Serum	Immunonephelometric
Insulin	INS	Serum	Immunochemiluminometric
Luteinizing hormone	LH	Serum	Immunochemiluminometric
Prolactin	PRL	Serum	Electrochemiluminescence immunoassay
Sex hormone–binding globulin	SHBG	Serum	Immunochemiluminometric
Testosterone	T	Serum	Immunochemiluminometric
Testosterone	T	Serum	Liquid chromatography/mass spectrometry
Thyroid-stimulating hormone	TSH	Serum	Immunochemiluminometric
Triglycerides	TRIGLY	Plasma	Photometric, enzymatic
Uric acid	UA	Plasma	Photometric, enzymatic
Stored biological samples			
			
Sample for DNA/RNA testing	Pax-gene	Plasma	N/A
			
Vaginal swab	eSwab/liquid amies	Vaginal wall	N/A

Abbreviation: IFCC, International Federation of Clinical Chemistry.

#### Menstrual cycle monitoring

The participants were offered opportunities to track their menstrual cycles after the study visits (dates of menstrual bleedings and ovulations, amount of bleeding), as well as different symptoms (pain and other discomforts, mood) related to their cycles, using the free Moona mobile application.^28^ The data collected via the application were downloaded and stored on the University of Oulu’s secure server.

#### Cognitive testing

Cognitive tests were planned and managed by Cambridge Cognition. This testing comprised 2 tests: the psychomotor vigilance task (PVT) and the emotional bias task (EBT). The PVT assessed how quickly participants reacted to stimuli, particularly touching a smartphone screen as soon as they saw a number. The EBT detected perceptual bias in facial emotion perception. Computer-morphed images derived from real people’s facial features were morphed between 2 emotions (eg, happy and sad) at varied intensities and presented to the participants one by one onscreen. The participants then indicated which emotion they thought each face displayed.

#### Data monitoring

Due to the study’s pharmaceutical collaboration (with Roche Diagnostics), part of the consent forms and data were monitored throughout the data collection process by an experienced, outsourced data monitoring company (Larix). Quality checks were performed for all collected data throughout the study by several staff members.

### Ethics approval

This study followed the principles of the Declaration of Helsinki. The ethics committees of the Northern Ostrobothnia and Helsinki and Uusimaa hospital districts approved this study (decision numbers 49/2019 and 483/2020, respectively). Our data use followed the European Union’s general data protection regulation (679/2016) and the Finnish Data Protection Act. All participants received written and oral information about the study and consented to participate in writing. The use of personal data is based on participants’ written, informed consent in their latest follow-up study, which may limit this use.

## Discussion

WENDY provides a unique data set that offers opportunities to evaluate lifelong reproductive and gynecological health and related factors among women. In this study, we focused on women in their mid-30s for several reasons. As women nowadays tend to postpone childbearing,^29^ many women in their mid-30s wish to conceive in the future. Hence, we found it especially important to investigate gynecological health in this specific age group. Moreover, many important gynecological diseases, such as PCOS and endometriosis, should have manifested by this age. The study’s strength is its extensive overview of fertile-aged women’s health with a variety of biological samples, measurements, interviews, and questionnaires. Additionally, the study’s participants are a representative, population-based sample of fertile-aged women in their mid-30s who were born in the same area. For over 80% of participants, follow-up data are available in the NFBC1986 data collection from the fetal stage onward. The NFBC1986 population is well documented, and a wealth of information from earlier data collection is already available. Almost all women (98%) who participated in WENDY also gave permission to link their data to national registries.^20^

This study also encountered some weaknesses. Of the total 5404 women invited to participate, 1918 (36%) participated. This participation rate aligns with the previous NFBC1986 clinical examinations.^21^ The greatest drawback to WENDY’s data collection was the COVID-19 pandemic and the subsequent lockdown in March 2020, only a week before the first scheduled data collection appointments. While the Oulu study site opened in May 2020, several peak COVID-19 outbreaks in Finland clearly affected study participation.^30^ Luckily, Finland’s restrictions during the pandemic were reasonable, and SARS-CoV-2 vaccinations were administered effectively. Moreover, we were able to collect unique data on how the COVID-19 pandemic affected different aspects of life among this female population. As attending the study required traveling to Oulu or Helsinki, some of the invited women had a long distance to both study sites, which may have restricted their ability to participate.

Additionally, participants’ life circumstances likely affected the study’s participation rate. Despite several reminder letters, this rate remained below 40%. Also, some women moved during the 2-year study period, and our letters failed to reach them. Moreover, since the invited women were of reproductive age, our instruction to postpone study visits until at least 6 months after childbirth may have restricted opportunities to participate. Notably, the NFBC participants were invited to WENDY shortly after they had already participated in NFBC1986 data collection in 2019-2020, which may have reduced their motivation to participate in another study. The WENDY participants from the NFBC1986 population were more educated than women who only participated in the NFBC1986 data collection but not in WENDY (basic or less 0.7% vs 1.2%, secondary 30.0% vs 40.1%, tertiary 69.3% vs 58.7%, *P* < 0.001). In Finland, education correlates with socioeconomical status and commonly also with health of the people.^31^ Finally, WENDY’s participants were almost entirely Caucasian, comprising an ethnically homogeneous population, which decreased the study’s intersubject variability but might limit our results’ generalizability to populations of different ethnicities.

A short email questionnaire was sent to participants 6 months after their data collection appointments to assess whether the study visits had influenced their health behavior. Most of the feedback received in response was very positive.

The study team is working on topics such as overall gynecological morbidity, PCOS, ovarian aging, female hyperandrogenism, contraceptive use, skin manifestations, pelvic floor strength, breast size evaluations, and interrectus distance analysis. We anticipate WENDY’s data to be highly published since, to date, more than 1700 scientific articles have been published on the NFBC data sets.^21^

## Data Availability

The WENDY data will be available upon request from the NFBC cohort center under certain conditions. The data can be requested from the NFBC cohort center, which will be directed to the WENDY research team. To support and encourage research collaborations, additional information can be found on the study’s webpage at https://www.oulu.fi/en/womens-health-study-wendy-protocol-population-based-study-assessing-gynecological-and-metabolic. All previously collected data of NFBC1986 can be found in the NFBC cohort catalog at https://www.oulu.fi/en/university/faculties-and-units/faculty-medicine/northern-finland-birth-cohorts-and-arctic-biobank/northern-finland-birth-cohorts.

## References

[1] Patla G, Mazur-Bialy AI, Humaj-Grysztar M, et al. Chronic vulvar pain and health-related quality of life in women with vulvodynia. *Life (Basel)*. 2023;13(2):328. 10.3390/life13020328 36836685 PMC9967635

[2] Della, Corte L, Di Filippo C, Gabrielli O, et al. The burden of endometriosis on women’s lifespan: a narrative overview on quality of life and psychosocial wellbeing. *Int J Environ Res Public Health*. 2020;17(13):4683. 10.3390/ijerph17134683 32610665 PMC7370081

[3] Li Y, Li Y, Yu Ng EH, et al. Polycystic ovary syndrome is associated with negatively variable impacts on domains of health-related quality of life: evidence from a meta-analysis. *Fertil Steril*. 2011;96(2):452-458. 10.1016/j.fertnstert.2011.05.072 21703610

[4] Gibson-Helm M, Teede H, Dunaif A, et al. Delayed diagnosis and a lack of information associated with dissatisfaction in women with polycystic ovary syndrome. *J Clin Endocrinol Metab*. 2017;102(2):604-612. 10.1210/jc.2016-2963 27906550 PMC6283441

[5] Ghai V, Jan H, Shakir F, et al. Diagnostic delay for superficial and deep endometriosis in the United Kingdom. *J Obstet Gynaecol*. 2020;40(1):83-89. 10.1080/01443615.2019.1603217 31328629

[6] Trutnovsky G, Plieseis C, Bjelic-Radisic V, et al. Vulvodynia and chronic pelvic pain in a gynecologic outpatient clinic. *J Psychosom Obstet Gynaecol*. 2019;40(3):243-247. 10.1080/0167482X.2018.1477753 29848143

[7] Hirode G, Wong RJ. Trends in the prevalence of metabolic syndrome in the United States, 2011-2016. *JAMA*. 2020;323(24):2526-2528. 10.1001/jama.2020.4501 32573660 PMC7312413

[8] Teede HJ, Misso ML, Costello MF, et al. Recommendations from the international evidence-based guideline for the assessment and management of polycystic ovary syndrome. *Fertil Steril*. 2018;33(9):1602-1618. 10.1093/humrep/dey256 PMC6112576 30052961

[9] Li B, Zhang Y, Zhang L, et al. Association between endometriosis and metabolic syndrome: a cross-sectional study based on the National Health and Nutrition Examination Survey data. *Gynecol Endocrinol*. 2023;39(1):2254844. 10.1080/09513590.2023.2254844 37673102

[10] Scottish Government . Women’s Health Plan: a plan for 2021-2024. Published August 20, 2021. Accessed November 7, 2023. https://www.gov.scot/publications/womens-health-plan

[11] Office of Research on Women’s Health . Perspectives on advancing NIH research to inform and improve the health of women. Updated January 3, 2022. Accessed November 8, 2023. https://orwh.od.nih.gov/sites/orwh/files/docs/ORWH-WHC-Report-508C.pdf

[12] Australian Government Department of Health and Aged Care . National Women’s Health Strategy 2020-2030. Published April 2019. Updated October 31, 2023. Accessed November 7, 2023. https://www.health.gov.au/resources/publications/national-womens-health-strategy-2020-2030

[13] World Health Organization . HRP annual report 2022. Published 2023. Accessed December 1, 2023. https://www.who.int/publications/i/item/9789240070684

[14] Department of Health and Social Care . Women’s health strategy for England. Published August 30, 2022. Accessed November 7, 2023. https://www.gov.uk/government/publications/womens-health-strategy-for-england/womens-health-strategy-for-england

[15] Womens Healthcare Market . Published January 2020. Accessed December 1, 2023. Accessed December 1, 2023. https://www.marketsandmarkets.com/Market-Reports/womens-health-care-market-136585329.html

[16] Colditz GA, Manson JE, Hankinson SE. The Nurses’ Health Study: 20-year contribution to the understanding of health among women. *J Womens Health*. 1997;6(1):49-62. https://doi:10.1089/jwh.1997.6.49 9065374 10.1089/jwh.1997.6.49

[17] Brown WJ, Dobson AJ. The Australian Longitudinal Study on Women’s Health: study design and sample. *N S W Public Health Bull*. 2000;11(1-2):3-4. 10.1071/NB00002 12105539

[18] Pergamit MR, Pierret CR, Rothstein DS, et al. The National Longitudinal Surveys. *J Econ Perspect*. 2001;15(2):239-253. 10.1257/jep.15.2.239

[19] El Khoudary SR, Greendale G, Crawford SL, et al. The menopause transition and women’s health at midlife: a progress report from the study of Women’s Health Across the Nation (SWAN). *Menopause*. 2019;26(10):1213-1227. 10.1097/GME.0000000000001424 31568098 PMC6784846

[20] Gissler M, Haukka J. Finnish health and social welfare registers in epidemiological research. *Norsk Epidemiologi*. 2004;4(1):113-120. https://doi:10.1093/ije/dyae092

[21] University of Oulu . University of Oulu: Northern Finland Birth Cohort 1986. Accessed April 5, 2023. http://urn.fi/urn:nbn:fi:att:f5c10eef-3d25-4bd0-beb8-f2d59df95b8e

[22] Järvelin MR, Elliott P, Kleinschmidt I, et al. Ecological and individual predictors of birthweight in a northern Finland birth cohort 1986. *Paediatr Perinat Epidemiol*. 1997;11(3):298-312. 10.1111/j.1365-3016.1997.tb00007.x 9246691

[23] Good Clinical Practice. Accessed May 4, 2023. https://gcp.nidatraining.org/

[24] Volløyhaug I, Mørkved S, Salvesen Ø, et al. Assessment of pelvic floor muscle contraction with palpation, perineometry and transperineal ultrasound: a cross-sectional study. *Ultrasound Obstet Gynecol*. 2016;47(6):768-773. 10.1002/uog.15731 26300128

[25] van Delft K, Thakar R, Sultan AH. Pelvic floor muscle contractility: digital assessment vs transperineal ultrasound. *Ultrasound Obstet Gynecol*. 2015;45(2):217-222. 10.1002/uog.13456 25044167

[26] Hohl A, Ronsoni MF, Oliveira MD. Hirsutism: diagnosis and treatment. *Arq Bras Endocrinol Metabol*. 2014;58(2):97-107. 10.1590/0004-2730000002923 24830586

[27] Ferriman D, Gallwey J. Clinical assessment of body hair in women. *J Clin Endocrinol Metab*. 1961;21(11):1440-1447. 10.1210/jcem-21-11-1440 13892577

[28] Korento . Mona application. Accessed February 13, 2023. https://www.facebook.com/korentory/photos/a.119176088163622/2820779268003277/?type=3

[29] Savelieva K, Jokela M, Rotkirch A. Reasons to postpone childbearing during fertility decline in Finland. *Marriage Fam Rev*. 2023;59(3):253-276. 10.1080/01494929.2022.2083283

[30] Coronavirus cases in Finland . Accessed February 15, 2023. https://www.thl.fi/episeuranta/tautitapaukset/coronamap.html

[31] OECD comparison: Socio-economic background still a strong influence on educational choices—regional variations in Finland are small . Published 2021. Accessed December 2, 2023. https://valtioneuvosto.fi/en/-/1410845/oecd-comparison-socio-economic-background-still-a-strong-influence-on-educational-choices-regional-variations-in-finland-are-small.

[32] Aalto A-M, Aro A, Teperi J. RAND-36 as a measure of health-related quality of life. Reliability, construct validity and reference values in the Finnish general population; 1999. Accessed February 5, 2023. https://www.julkari.fi/handle/10024/76006

[33] Hayes R, Sherbourne C, Mazel R, et al. The RAND 36-item health survey 1.0. *Health Econ*. 1993;2(3):217-227. 10.1002/hec.4730020305 8275167

[34] Stunkard AJ, Sørensen T, Schulsinger F, et al. Use of the Danish adoption register for the study of obesity and thinness. *Res Publ Assoc Res Nerv Ment Dis*. 1983;60:115-120. 6823524

[35] Jackson KL, Janssen I, Appelhans BM, et al. Body image satisfaction and depression in midlife women: the Study of Women’s Health Across the Nation (SWAN). *Arc Womens Ment Health*. 2014;17(3):177-187. 10.1007/s00737-014-0416-9 PMC4026204 24623160

[36] Littleton HL, Axsom D, Pury CLS. Development of the body image concern inventory. *Behav Res Ther*. 2005;43(2):229-241. 10.1016/j.brat.2003.12.006 15629752

[37] Witting K, Santtila P, Jern P, et al. Evaluation of the female sexual function index in a population based sample from Finland. *Arch Sex Behav*. 2008;37(6):912-924. 10.1007/s10508-007-9287-8 18335306

[38] Carpenter JS, Jones SMW, Studts CR, et al. Female sexual function index short version: a MsFLASH item response analysis. *Arch Sex Behav*. 2016;45(8):1897-1905. 10.1007/s10508-016-0804-5 27502350 PMC5053877

[39] Isidori AM, Pozza C, Esposito K, et al. Development and validation of a 6-item version of the female sexual function index (FSFI) as a diagnostic tool for female sexual dysfunction. *J Sex Med*. 2010;7(3):1139-1146. 10.1111/j.1743-6109.2009.01635.x 19968774

[40] Borud EK, Martinussen M, Eggen AE, et al. The Women’s Health Questionnaire (WHQ): a psychometric evaluation of the 36-item Norwegian version. *Scand J Psychol*. 2009;50(2):183-189. 10.1111/j.1467-9450.2008.00701.x 19170970

[41] Elbatarny M, Mollah S, Grabell J, et al. Normal range of bleeding scores for the ISTH-BAT: adult and pediatric data from the merging project. *Hemophilia*. 2014;20(6):831-835. 10.1111/hae.12503 PMC4251588 25196510

[42] Beer GM, Schuster A, Seifert B, et al. The normal width of the linea alba in nulliparous women. *Clin Anat*. 2009;22(6):706-711. 10.1002/ca.20836 19637295

[43] van de Water AT, Benjamin DR. Measurement methods to assess diastasis of the rectus abdominis muscle (DRAM): a systematic review of their measurement properties and meta-analytic reliability generalisation. *Man Ther*. 2016;21:41-53. 10.1016/j.math.2015.09.013 26474542

